# Adolescent hallux valgus: a systematic review of outcomes following surgery

**DOI:** 10.1007/s11832-015-0655-y

**Published:** 2015-04-22

**Authors:** Ziad Harb, Michail Kokkinakis, Hiba Ismail, Gavin Spence

**Affiliations:** Evelina London Children’s Hospital, Guy’s and St Thomas’ NHS Foundation Trust, Westminster Bridge Road, London, SE1 7EH UK; Stanmore Royal National Orthopaedic Hospital, London, UK

**Keywords:** Hallux valgus, Adolescent, Paediatric, Bunion, Metatarsus

## Abstract

**Purpose:**

The management of adolescent hallux valgus (AHV) remains controversial, with reservations about both conservative and surgical treatments. Non-operative management has a limited role in preventing progression. Surgical correction of AHV has, amongst other concerns, been associated with a high prevalence of recurrence of deformity after surgery. We conducted a systematic review to assess clinical and radiological outcomes following surgery for AHV.

**Methods:**

A comprehensive literature search was performed in the Cochrane Library, CINAHL, EMBASE, Google Scholar and PubMed. The study was performed in accordance with the recommendations of the Preferred Reporting Items for Systematic Reviews and Meta-Analyses (PRISMA) guidelines. Demographic data, radiographic parameters and results of validated clinical scoring systems were analysed.

**Results:**

The published literature on AHV is largely heterogeneous and retrospective. Nine contemporary studies reporting on 140 patients (201 osteotomies) were included. The female to male ratio was 10:1. The mean age at operation was 14.5 years (range 10.5–22). The mean follow-up was 41.6 months (range 12–134). The mean post-operative American Orthopaedic Foot and Ankle Society (AOFAS) score was 85.8 (standard deviation, SD ±7.38). The mean AOFAS patient satisfaction showed that 86 % (SD ±11.27) of patients were satisfied or very satisfied with their outcome. On the duPont Bunion Rating Score (BRS), 90 % rated their outcome as good or excellent. There was a statistically significant improvement in the inter-metatarsal angle (IMA, *p* = 0.0003), hallux valgus angle (HVA, *p* < 0.0001) and distal metatarsal articular angle (DMAA, *p* = 0.019).

**Conclusion:**

Based on the most current published evidence, contemporary surgical interventions for AHV show excellent clinical and radiological outcomes, with high patient satisfaction. The rates of recurrence and other complications are lower than the historically reported figures. There is a need for high-level, multi-centre collaborative studies with prospective data to establish the long-term outcomes and optimal surgical procedure(s).

## Introduction

Hallux valgus is a common condition and has been reported to affect up to 36 % of the paediatric population [1]. The exact aetiology of adolescent hallux valgus (AHV) is unknown, but several features have been identified as possible predisposing factors to its development. These include a positive family history (usually maternal), female gender, pes planus, a relatively long first metatarsal, constrictive footwear and metatarsus primus varus [2, 3]. Symptoms include a painful, erythematous bunion, clinical deformity and unsatisfactory cosmesis, and difficulty finding appropriate footwear.

The treatment of AHV is controversial, since there are reservations about both conservative and surgical treatments. Non-operative management is usually based on footwear modifications, orthotics and analgesia, but has been shown to have a limited role in preventing progression [4]. Surgical correction of AHV is often indicated once conservative treatment has failed; however, there are over 130 surgical procedures described for hallux valgus, indicating that there is not one procedure that is preferred [5]. Also, AHV has traditionally been associated with a high prevalence of recurrence of deformity after surgery [6, 7], with reports of up to 61 % recurrence rates in one study [8].

With the aim of assessing the results of surgery for hallux valgus in the paediatric population, we conducted a systematic review to evaluate the published literature on clinical and radiological outcomes, complication rates and recurrence following AHV correction. To the best of our knowledge, this is the first systematic review on AHV surgery in the published literature.

## Methods

### Search strategy and criteria

The Cochrane Library, CINAHL, EMBASE, Google Scholar and PubMed electronic databases were searched for all relevant articles. The bibliographies of the retrieved articles were further examined for additional relevant articles.

Each database was searched from its inception date up until July 2014. The search terms and Booleans used are summarised in Table 1. All articles that met the pre-defined inclusion criteria were included. The inclusion criteria were as follows: case series, cohort studies or clinical trials on surgical outcomes for the correction of AHV; follow-up for a minimum of 12 months; clinical outcomes using internationally recognised and validated outcome measures; and basic patient demographics within the body of the paper. Exclusion criteria comprised any paper that did not meet the inclusion criteria, as well as those that included patients with significant co-morbidities, such as rheumatoid disease or an underlying neuromuscular disorder, or those not published in the English language.

**Table 1:** The search terms and Booleans used in the retrieval of relevant articles from the electronic databases

Search term and Boolean
(hallux valgus[Title]) AND adolescent[Title]
(paediatric[Title]) AND hallux valgus[Title]
(pediatric[Title]) AND hallux valgus[Title]
(juvenile[Title]) AND hallux valgus[Title]
(juvenile[Title]) AND metatarsus adductus[Title]
(juvenile[Title]) AND metatarsus primus varus[Title]
(juvenile[Title]) AND bunion[Title]
(adolescent[Title]) AND bunion[Title]
(adolescent[Title]) AND metatarsus varus[Title]
(adolescent[Title]) AND metatarsus adductus[Title]
(pediatric[Title]) AND metatarsus adductus[Title]
(pediatric[Title]) AND metatarsus varus[Title]
(children[Title]) AND metatarsus varus[Title]
(children[Title]) AND hallux valgus[Title]

### Data collection and analysis

The study was performed in accordance with the recommendations of the Preferred Reporting Items for Systematic Reviews and Meta-Analyses (PRISMA) group [9]. Two authors (ZH, MK) performed the literature searches and reviewed the abstracts, and articles deemed to meet the inclusion criteria were retrieved and reviewed fully. In the event of any discrepancy between the two authors, opinion was referred to a third author (GS) for resolution. Demographic data were collected, including the number of patients, number of feet treated, male to female ratio, age and length of follow-up. Data were also collected on the type of scoring system used, its results and radiological parameters, such as the hallux valgus angle (HVA), inter-metatarsal angle (IMA) and distal metatarsal articular angle (DMAA). A record was made of all reported complications and cases of recurrence. Data were extracted from the papers by systematic analysis of each article and summarisation in Microsoft Excel version 2010 (Microsoft, Redmond, WA, USA). Statistical analysis was performed using RevMan 5 (The Nordic Cochrane Centre, The Cochrane Collaboration 2009, Copenhagen, Denmark) and SPSS version 20 (IBM, New York, NY, USA).

## Results

### Study characteristics

**Fig. 1:**
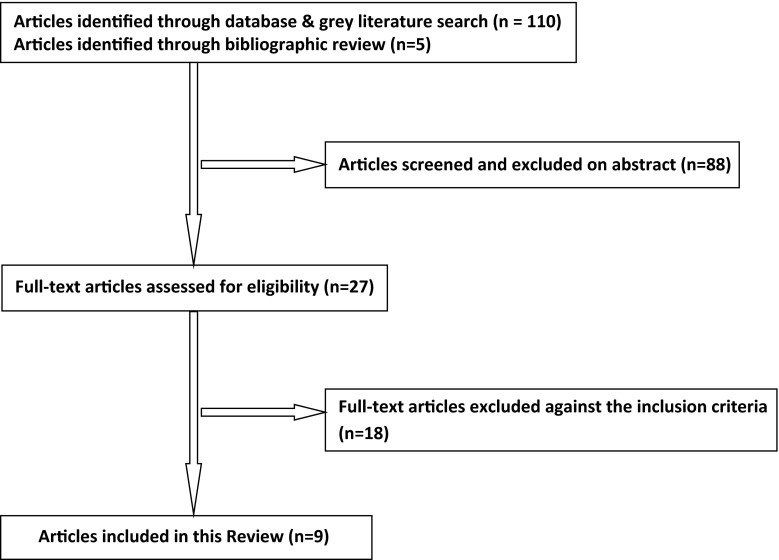
Preferred Reporting Items for Systematic Reviews and Meta-Analyses (PRISMA) diagram of the search results

See Fig. 1 for the PRISMA diagram of the search results. In total, 115 papers were retrieved from the initial search strategy. Nine studies met the inclusion criteria and are included in this study [10–18].

From the nine studies, 201 corrective osteotomies were performed on 142 patients. There were 129 females and 13 males, giving a female to male ratio of 9.9:1. The mean age at operation was 14.5 years (range 10.5–22). The mean follow-up was 41.64 months (range 12–134). Six of the nine studies used the American Orthopaedic Foot and Ankle Society (AOFAS) score [19], three used the duPont Bunion Rating Score (BRS) [1], one used the American College of Foot and Ankle Surgeons (ACFAS) score [20] and one utilised the Japanese Society for Surgery of the Foot (JSSF) scoring system [21].

Only one paper [10] included any pre-operative clinical outcomes. All nine articles measured pre- and post-operative HVA and IMA. Two articles [11, 18] did not measure the DMAA.

### Clinical diversity

Numerous surgical techniques have been described to correct hallux valgus in the general population, and this considerable diversity is also reflected in the paediatric cohort [5]. In the nine articles included in this study, three used the scarf first metatarsal osteotomy [13–15]; two used a proximal metatarsal osteotomy [10, 16]; one chevron distal metatarsal osteotomy [11]; one double metatarsal osteotomy [17]; one modified Simmonds–Menelaus procedure (whereby the bone fragment from the bunionectomy is used as a graft inserted into a proximally based first metatarsal opening wedge osteotomy without any internal fixation) [18]; one percutaneous technique for achieving correction, whereby a distal osteotomy and bunionectomy were performed through a small stab incision medially, a lateral soft-tissue release using a Beaver blade via a laterally based incision and a proximal phalanx wedge osteotomy via a second medial incision; finally, if the IMA was greater than 18°, a dorsal wedge osteotomy at the base of the metatarsal was performed through a dorsal approach [12]. Of the 201 operated feet, only 19 (9.5 %) were reported to have also had a proximal phalanx closing wedge (Akin) osteotomy.

Of the nine papers, three [14, 15, 17] did not specifically mention whether they performed any soft-tissue procedures to augment their bony corrections. Six articles did describe additional soft-tissue procedures, but there was heterogeneity between the papers’ techniques. Gicquel et al. [12] performed releases of the sesamoid-phalangeal and sesamoid-metatarsal ligaments, whereas Andreacchio et al. [18] released the adductor tendon and re-sutured it to the metatarsal head, as well as a transverse metatarsal ligament release and capsular shortening. Okuda et al. [10] also released the adductor tendon and transverse metatarsal ligament, but the capsule was plicated with the abductor tendon. The remaining three articles [11, 13, 16] released the adductor tendon and performed a capsulorrhaphy.

There was also variation in the initial post-operative management, with three of the nine studies having no documentation on their post-operative mobilisation; the other six studies also showed heterogeneity in their weight-bearing status and method of immobilisation. The results are summarised in Table 2.

**Table 2:** Summary of the included studies

References	No. of patients (no. of feet)	Follow-up in months (range)	Patient age (range)	F:M ratio	Scoring system	Radiographic measures	Post-op plan
Okuda et al. [10]	11 (12)	22 (12–36)	17 (13–22)	11:0	JSSF	HVA, IMA	2/52 NWB cast, 1/52 PWB cast, FWB shoe with arch support
Kraus et al. [11]	12 (15)	84.3 (2.2–11.2)	14.7 (11.7–17.3)	10:2	AOFAS	HVA, IMA	6/52 FWB cast
Gicquel et al. [12]	18 (33)	31.5 (14.1–58.2)	<16	18:0	AOFAS	HVA, IMA, DMAA	FWB, 6/52 bandage to medialise hallux, 6/12 1st webspace toe spacer
Farrar et al. [13]	29 (39)	38.6 (6–60)	14.1 (10–17)	29:0	AOFAS	HVA, IMA, DMAA	6/52 heel WB shoe
John et al. [14]	7 (14)	57	14.4 (12–17	6:1	AOFAS, ACFAS	HVA, IMA, DMAA	Not recorded
George et al. [15]	13 (19)	37.6 (22.5–76.3)	14.3 (12–18)	11:2	AOFAS	HVA, IMA, DMAA	Not recorded
Petratos et al. [16]	32 (39)	42 (32–62)	14.2 (11.8–15.3)	27:5	duPont BRS	HVA, IMA	6/52 cast (WB status not recorded)
Johnson et al. [17]	9 (10)	27	15 (13–17)	6:3	AOFAS, duPont BRS	HVA, IMA, DMAA	Not recorded
Andreacchio et al. [18]	11 (20)	34.8 (26.4–49.2)	12.4 (10.5–14.5)	11:0	duPont BRS	HVA, IMA	3/52 NWB cast

*JSSF* Japanese Society for Surgery of the Foot, *AOFAS* American Orthopaedic Foot and Ankle Society, *ACFAS* American College of Foot and Ankle Surgeons, *duPont BRS* duPont Bunion Rating Score, *HVA* hallux valgus angle, *IMA* inter-metatarsal angle, *DMAA* distal metatarsal articular angle, *NWB* non-weight-bearing, *PWB* partial weight-bearing, *FWB* full weight-bearing, *WB* weight-bearing

### Outcome analysis: clinical outcomes

Six papers [11–15, 17] used the AOFAS score for measuring clinical outcomes, of which none provided any pre-operative scores. The mean post-operative AOFAS score was 85.8 (standard deviation, SD ±7.38; range 54–100). Of these six papers, three provided patient satisfaction scores, with 92 % [11], 73 % [12] and 93 % [13] of patients being satisfied or very satisfied at a mean final follow-up of 83.4, 31.5 and 38.6 months, respectively, giving a mean of 86 % (SD ±11.27) of patients satisfied or very satisfied with their outcome.

On the duPont BRS, three studies [16–18] reported on a total of 69 feet (50 patients). In total, 19/69 (28 %) rated their outcome as excellent, 43/69 (62 %) as good, 6/69 (9 %) as fair and 1/69 (1 %) rated their results as poor.

As well as the AOFAS score, one paper [14] also used the ACFAS score, and observed a mean score of 94.7 (range 57–100) in seven patients (14 feet).

One study [10] that utilised the JSSF scoring system showed an improvement of the mean pre-operative score of 62 (49–75) to 99.2 (90–100) (*p* = 0.002).

### Outcome analysis: radiological outcomes

See Table 3 for a summary of the main findings and Table 4 for a breakdown of the individual results from each included paper.

**Table 3:** Summary of radiological measurements

	HVA		IMA		DMAA	
	Pre-op	Post-op	Pre-op	Post-op	Pre-op	Post-op
Mean (°)	30.05	15.58	16.69	9.75	17.26	11.01
Standard deviation	6.20	4.86	7.3	5.21	3.89	4.19
Range (°)	15.3–35.8	8–25	13.2–36	5.64–22	12.8–24.5	6.6–16.9

**Table 4:** Summary of individual results from the included papers

Paper	Cases (*n*)	HVA					IMA					DMAA				
		Pre-op	SD	Post-op	SD	95 % CI	Pre-op	SD	Post-op	SD	95 % CI	Pre-op	SD	Post-op	SD	95 % CI
Okuda et al. [10]	12	32.3	–	12.2	–	–	14	–	6.2	–	–	16.9	–	16.9	–	–
Kraus et al. [11]	15	31.5	8.6	14.4	4.6	27.2–35.9/12.1–16.7	13.2	2.1	6.1	2.1	12.1–14.3/5–7.2	–	–	–	–	–
Gicquel et al. [12]	33	28.06	6.3	19.45	8.52	25.9–30.2/16.5–22.4	13.61	2.59	12.74	2.7	12.7–14.5/11.8–13.7	15.97	5.74	8.97	8.17	14–17.9/6.2–11.8
Farrar et al. [13]	39	34.8	–	16.3	–	32.9–36.7/14–18.6	15.9	–	8.8	–	15–16.8/8–9.6	16	–	9.2	–	14.5–17.5/7.7–10.7
John et al. [14]	14	27.53	8.53	12.79	6.74	23–31.9/9.3–16.3	14.29	2.6	5.64	2.29	12.9–15.7/4.4–6.8	24.5	9.11	8.79	6.76	19.7–29.3/5.3–12.3
George et al. [15]	19	34	–	25	–	–	14	–	8.5	–	–	17.4	–	15.6	–	–
Petratos et al. [16]	39	15.3	–	8	–	–	36	–	22	–	–	–	–	–	–	–
Johnson et al. [17]	10	35.79	–	14.25	–	–	15.75	–	6.5	–	–	12.8	–	6.6	–	–
Andreacchio et al. [18]	20	31.2	–	17.8	–	–	13.5	–	11.3	–	–	–	–	–	–	–

**Fig. 2:**
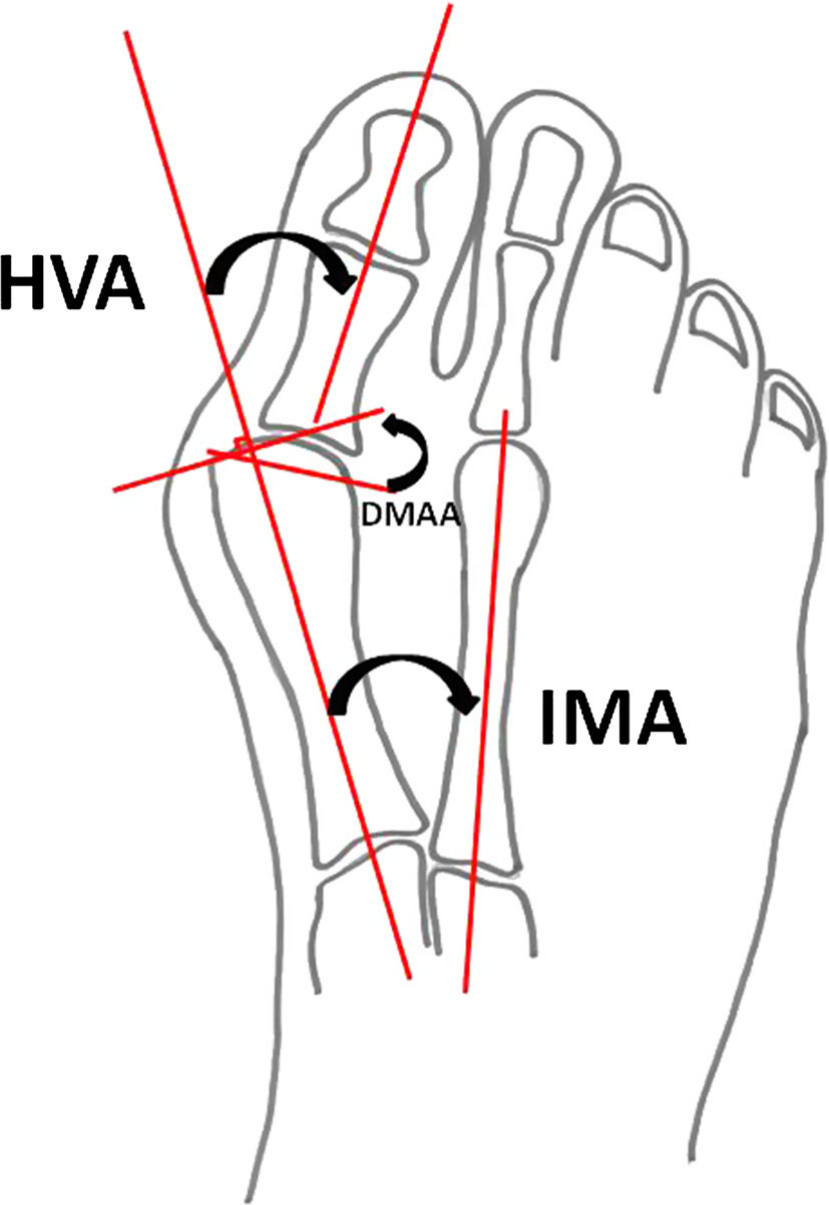
The radiographic angles commonly used for hallux valgus. *HVA* hallux valgus angle (10°–15°), *IMA* inter-metatarsal angle (7°–9°), *DMAA* distal metatarsal articular angle (<8°)

The normal values [15] for the radiological angles that are commonly measured are: IMA 7°–9°, HVA 10°–15° and DMAA <8°. These are graphically demonstrated in Fig. 2.

From all nine studies, the mean pre-operative IMA was 16.7° (SD ±7.3, range 13.2–36) and the mean post-operative IMA was 9.8° (SD ±5.21, range 5.6–22) (*p* = 0.0003). The mean pre-operative HVA improved from 30.1° (SD ±6.2, range 15.3–35.8) to 15.6° (SD ±4.86, range 8–25) (*p* < 0.0001). From the six studies that measured the DMAA, there was an improvement from 17.3° (SD ±3.89, range 12.8–24.5) to 11.01° (SD ±4.19, range 6.6–16.9) (*p* = 0.019).

If subgroup analysis is performed on the three studies that used the scarf osteotomy, the HVA improved from 32.1° (SD ±3.99, range 27.5–34.8) to 18° (SD ±6.29, range 12.8–25) (*p* < 0.0001), the IMA improved from 14.7° (SD ±1.02, 14–15.9) to 7.6° (SD ±1.74, 5.6–8.8) (*p* < 0.0001) and the DMAA was corrected from a mean pre-operative angle of 19.3° (SD ±4.56, 16–24.5) to a mean post-operative angle of 11.2° (SD ±3.8, 8.8–15.6) (*p* < 0.0001).

### Outcome analysis: complications

Out of a total of 201 feet, there were 4 (2 %) cases of infections.

There were 24 (11.9 %) cases of significant post-operative pain. A total of 9 feet (4.5 %) had scar hypersensitivity.

Recurrence of deformity was encountered in 16 cases (8 %). The overall revision rate was 4 %, representing eight cases, although the indication for revision was only stated in two cases, one for non-union and the other for recurrence 18 years after the primary procedure.

There was 1 (0.5 %) case of non-union, 1 (0.5 %) metatarsalgia, 1 (0.5 %) foot developed complex regional pain syndrome (CRPS) and one case where the patient was dissatisfied with the cosmetic appearance. There were no reports of avascular necrosis of the metatarsal head.

There were 20 (10 %) feet that had an under-correction; however, these were all in the same paper, which used a percutaneous approach to correct deformity [12].

Out of a total of 201 feet, 85 (42.3 %) had a reported complication. This is somewhat skewed by the one study that used a percutaneous approach to correct deformity, and accounted for 39 complications. Thus, excluding this study, the overall complication rate is 46/201 (22.9 %).

### Methodological analysis

**Fig. 3:**
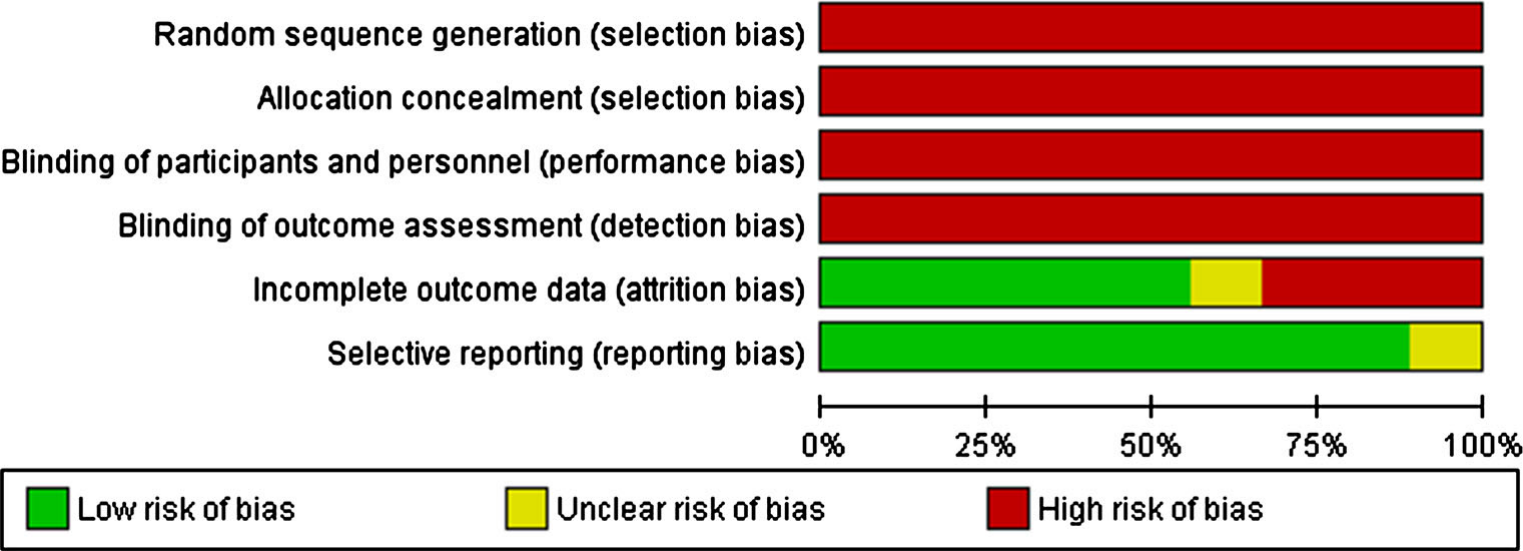
Risk of bias graph: review authors’ judgements about each risk of bias item presented as percentages across all included studies

**Fig. 4:**
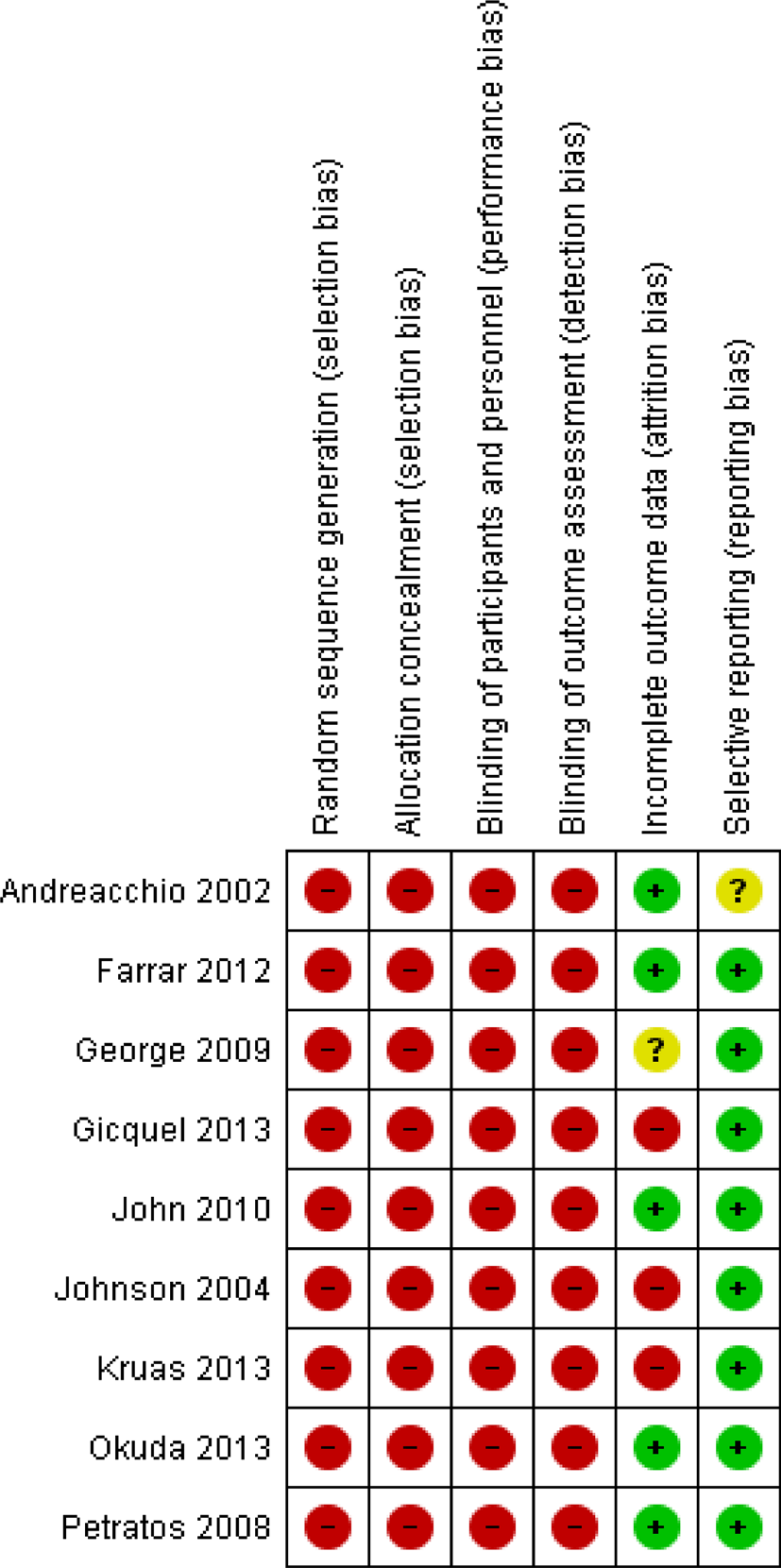
Risk of bias summary: review authors’ judgements about each risk of bias item for each included study

The results of the assessment of the risk of bias are shown in Figs. 3 and 4.

All nine articles were case series, and only one was a prospective study [10]; the remaining eight studies were retrospective. There is, therefore, a high risk of patient selection bias, as well as performance bias. As they were all case series, there is no blinding or randomisation of patient selection in any of the studies included in this review.

## Discussion

AHV is a common foot disorder that can cause significant pain, difficulty with footwear and cosmetic dissatisfaction. Traditionally, surgery to correct AHV has been associated with poor outcomes, often due to multiple factors, including sub-optimal correction, excessive first metatarsal shortening, inadequate fixation, non-union and recurrence. However, as techniques and methods of fixation have improved, the outcomes have evolved and the aim of this review was to assess the published data in this patient population to establish the clinical and radiological outcomes.

This study suggests that, based on current published evidence, overall, surgical treatment for AHV using the described surgical techniques in the included articles has excellent clinical and radiological results, with a high majority of patients being satisfied or very satisfied with their outcome. The post-operative AOFAS score ranged from 80 to 96.4 %. Radiological parameters also improved significantly, although it has been shown that pre-operative radiographic angles are not reliable predictors of clinical outcome and patient satisfaction [13]. And, although the radiological angles were improved, they were not always necessarily corrected to within the normal range.

Excluding the one study that utilised a percutaneous technique for achieving correction, the overall complication rate was 22.9 %. The recurrence rate was 8 %, and since the included studies are all contemporary, this represents a lower rate compared to the historical figures that often quoted high recurrence rates of up to 61 % [17]. The high recurrence rate is often attributed to the fact that, unlike in the adult population, the first metatarsal physis is still open [18] and, therefore, it is usually preferred to delay AHV surgery until mid- to late teens to allow the physis to close and, thus, reduce the risk of recurrence. This is reflected in the included articles, where the mean age at operation was 14.5 years. Whilst there are difficulties in establishing whether the physis has closed or not, as well as the variability at which this happens, one study [13] did divide their cohort into patients aged 10–14 years and those aged 14–17 years. The recurrence rate for the younger group was 3/22 and for the older group 4/17, and, although this is a small single-centre case series, it would suggest that an open physis is not necessarily the main contributing factor to the recurrence risk. Further studies are needed in order to establish whether delaying surgery for AHV until skeletal maturity is advantageous and has better outcomes.

Our study has several limitations due to the nature of the included articles for review. All of the studies were small case series from single centres. All but one study [10] were retrospective in nature. There is no attempt at randomisation or blinding, and, as such, these papers are open to selection, performance and detection bias. Only one of the included studies recorded pre-operative clinical outcomes [10], thus making it difficult to ascertain the true impact of the effectiveness of the surgical procedure. Also, there was heterogeneity in the surgical technique used for achieving correction of the deformity; three studies focused on the scarf osteotomy and subgroup analysis shows statistically significant improvements in clinical and radiological outcomes following this procedure. Cadaveric biomechanical studies support these clinical findings and have demonstrated that the scarf osteotomy is more stable than distally based osteotomies under physiological loading [22, 23]. It is also difficult to draw any firm conclusions about which surgical technique provides the optimum clinical and radiological outcomes because of the variety of techniques used, meaning that comparison studies would rely on single case series only. Interestingly, 17 of the 19 Akin osteotomies were performed on patients that had undergone a scarf osteotomy. This may be due to surgeon preference, but it may also reflect the fact that the scarf osteotomy alone tended to under-correct the deformity.

There were also inconsistencies in the reporting of the post-operative management plans and variation in those that did report their post-operative mobilisation status; there is limited evidence to support one particular regimen over another, although one biomechanical study did show that there was no difference in the outcomes of patients with normal bone stock when managed with different types of immobilisation [23].

Another limitation is the weakness in the level of data reporting of the included studies, such that certain statistical analysis was impossible. Only four papers provided basic descriptive statistics such as standard deviations or confidence intervals.

## Conclusion

The current published literature on surgical outcomes for primary adolescent hallux valgus (AHV) is of low quality of evidence, and there is a need for high-level, multi-centre collaborative studies with prospective data and larger sample sizes, incorporating patient-focused outcome measures, as well as the internationally validated outcome scores. Based on the limited available evidence, current treatment with scarf osteotomy, as well as basal osteotomies, show very good clinical and radiological outcomes, and high patient satisfaction. Crucially, the recurrence rates are lower than the traditionally quoted figures, and this may impact on our threshold for referring for surgery. The current evidence base does not allow for a significant comparison between different surgical techniques to give a meaningful insight into which technique offers the most superior outcomes.
